# A recommended management plan for coronavirus disease 2019–positive geriatric patients based in South African old age homes

**DOI:** 10.4102/safp.v63i1.5222

**Published:** 2021-02-08

**Authors:** Christina Eleftheriades

**Affiliations:** 1 Department of Family Medicine, Faculty of Health Sciences, University of the Witwatersrand, Johannesburg, South Africa

**Keywords:** family medicine, COVID-19, frail care, transitional care, geriatrics

## Abstract

The coronavirus disease 2019 (COVID-19) pandemic has had a profound impact on elderly patients, and thus, adequate treatment plans are essential. This qualitative report provides recommendations for the supportive care and treatment of residents in long-term care facilities (LTCF) with COVID-19. A treatment protocol was developed in response to an outbreak of COVID-19 in an LTCF based in Johannesburg and was implemented over a 3-month period.

The Rand Aid Association (RAA) is a non-profit organisation comprising of retirement villages and frail cares in Johannesburg, Gauteng, offering accommodation to approximately 3000 aged people. The main frail care within the RAA is the Ron Smith Care Centre (RSCC) situated in Lyndhurst, Gauteng. It comprises of six wards, approximately 130 residents ranging in ages of 46–103 years old and the vast majority between 85 and 90 years old. The latter residents have multiple co-morbidities with a score greater than three on the frail scale^1^ and have some degree of neurocognitive impairment ranging from mild to severe. A dedicated multidisciplinary team supports and cares for the residents of the frail care. They include 40 nursing staff per shift (including carers, enrolled nurses, staff nurses and registered nurse), matron, occupational therapists, social workers, dietician, dentist, audiologist, speech therapist, biokineticist, podiatrist, physiotherapist and two doctors trained in geriatric care.

On 02 June 2020, the first resident tested positive for coronavirus disease 2019 (COVID-19). A geriatric peer-reviewed meeting was held between the two general practitioners (both having completed their diploma in geriatric medicine) and a team of sub-specialist geriatricians. A care protocol was developed to manage COVID-19-positive patients within the long-term care facilities (LTCF) and to improve morbidity and mortality and more so lessen the need for hospital admissions (Table 1-A1).^2^

Preventative measures prior to the national lockdown were implemented and included the following: restriction of visitors and non-essential staff; screening of staff via twice-daily measurement of body temperature; COVID-19 symptom monitoring of residents and staff; personal protective equipment (PPE) to be worn by staff including mask, apron, visor and gloves; social distancing; and the restriction of any social gatherings such as occupational therapy and biokinetics.^3^

In addition, many of the residents had pre-existing advanced directives and advanced care planning, before the onset of the pandemic. A multidisciplinary team was established prior to the lockdown comprising of social workers and nursing staff. The team educated families and counselled them on the worldwide trends of the outbreak and the poor outcomes of the elderly with regard to a ‘realistic expectations’ and the need for supportive care. The vast majority of families, from the outset, requested that their positive relatives not be hospitalised.

## Measures for monitoring residents

Other clinical parameters measured were 12-hourly blood pressure (BP), pulse rate (PR) and temperature. The parameters had to be interpreted in unison, bearing in mind that temperature is not an accurate measure of infection in the older patient.^4^ Regular saturation monitoring helped identify patients in respiratory distress and in need of supplemental oxygen. Studies have shown that nasal prong oxygen (NPO) was the preferred method of respiratory support for patients in acute respiratory distress syndrome with COVID-19.^5^

The clinical condition in older patients can change within a day, and studies amongst older patients have identified delirium as a sign of acute illness.^6^ The nursing staff within the LTCF are dementia trained staff. They are also permanent staff, rather than contractually based, and thus, they are trained to identify delirium with underlying dementia.

## The first positive case

Positive residents were immediately removed from the ward and placed in an ad-hoc isolation unit within the frail care. The aim was to keep the other patients negative and protect the staff.

## Treatment plan

The treatment plan was based around identifying delirium and monitoring saturation, respiratory rate (RR), PR and temperature. Residents were monitored 6–8 hourly and readings charted. Hypoxic residents were put immediately onto NPO. Residents were started at a rate of 2 L/min and flow titrated up to 5 L/min with the aim of maintaining oxygen saturation above 92%. Haematological tests monitored full blood count (FBC); identified lymphopaenia, C-reactive protein (CRP), the necessity for either intramuscular antimicrobial therapy or hospital admission, and D-dimer >10 which occurred towards the latter part of the disease; demonstrated a poor prognostic outcome; and identified urea and creatinine hydration status in patients with advanced dementia.

The acute medical treatment was based on accuhalers, in particular synchronised breathing inhalers. The accuhalers helped maintain oxygen saturation without the use of nebulisation. If a patient complained of chest tightness or decompensation noted on pulse oximetry, then a per-patient decision was made to give intramuscular cortisone.^7,8^

With regard to antimicrobials, the first-line treatment of choice was azithromycin.^9^ In some residents with moderate to severe disease, where possible bacterial secondary infection was suspected, ceftriaxone was administered either intravenously or intramuscularly (diluted with lignocaine).

Cardiorespiratory physiotherapy focused on respiratory treatment of our patients with COVID-19. Physiotherapists were encouraged to help educate our patients on cough etiquette. Sputum induction, nebulisation, suctioning and other aerosol generating procedures were not permitted.^10^ Coronavirus disease 2019 led to prolonged immobilisation, reduced muscle function and eventual sarcopenia. Physiotherapists therefore helped with early mobilisation of frail patients with prone positioning over a chair or, siting (edge of bed) with an array of basic exercises.

In order to prevent COVID-19 vasculitis, a type 3 hypersensitivity (immune complex disease) response,^11^ patients were placed on an empiric anticoagulant.^12^ The anticoagulants used were either low molecular weight heparin or rivaroxiban 10 mg daily. Patients were kept on rivaroxiban for a period of approximately 30 days. Further, anti-inflammatory and thrombolytic properties were identified with the use of statins^13^ and later on in the course of the pandemic, colchicine.

## Referral to hospital

Social parameters for hospital admission were either an absent advanced directive or families request. Medical parameters for hospital admission were oxygen saturation levels not maintaining at 90% on 5 L NPO and the LTCF not having a high-flow system. Haematological parameters for hospital admission were CRP > 100 after oral antibiotics, D-dimer of > 10, acute kidney injury (dehydration), chronic delirium and patients with severe underlying chronic co-morbidities such as dialysis.

Financial constraints forced families to opt for hospital admissions in order to access ‘in-hospital’ benefits, even for palliative purposes. The medical aids would not cover the costs incurred by the LTCF to treat the older patients out-of-hospital with regard to phlebotomy, medication and physiotherapy.

## Results of the protocol

One hundred and thirty-six residents were there in the RSCC at the time of outbreak on 02 June 2020. Over a 3-month period, 61 residents tested positive, 23 died and 9 were admitted to hospital. The majority of fatalities were residents of the advanced dementia wing.

### Insights gained on preventative measures

With regard to staff, preventative measures need to be repeatedly enforced. The wing shift leaders were key in reminding and constantly re-iterating the importance of PPE. Disposable gloves and aprons were available outside every room. Hand sanitiser was filled daily by the cleaning staff, and dispenser sets were placed at the entrance of all wings. Posters were placed on the noticeboards and along the passageways as a constant visual reminder to wear PPE and sanitise.

With regard to the residents, high-protein nutritional supplementation was necessary to reduce complications such as bed sores, other infections and delayed recovery.^14^ In addition, vitamin A, B, D and zinc as a combination, effervescent daily multivitamin supplement with breakfast helped enhance viral immunity.^15^ Practically, effervescent formulations worked better with our patients, particularly in those with dysphagia in advanced dementia.

### Insights gained on monitoring positive residents

Monitoring resident’s oxygen saturation and vitals required an oxygen saturation monitor and working sphygmomanometer in each wing. The readings had to be correctly charted and interpreted by either the doctor or the sister in charge of the wing. This led to human error and often charts were missing, misplaced or illegible. Perhaps, a robust paperless system would be more reliable. Furthermore, the task of capturing clinical notes and documentation of patient history is fraught with the risk of contamination. The idea of a ‘clean zone’ for making notes after the rounds should be considered. In order to communicate with patients and residents in the isolation ward, using walkie-talkie is easier than cell phone communication. All healthcare workers were required to have their names clearly written on their aprons so that they were identifiable to residents and themselves. Also, patients with advanced dementia related better to the ‘named’ PPE-clad healthcare workers.

Delirium is an important indicator of sepsis in the older patient and formed the basis of daily vitals. Educating staff on the importance of identifying delirium and notifying the doctors when there was delirium had to be emphasised. There are various tests available to identify delirium, and perhaps, offering seminars to the staff on the signs of delirium rather than signs of COVID in an LTCF maybe more helpful. Finally, educating the nursing staff on the importance of tachypnoea in COVID as a poor prognostic indicator and on accurate measurement of a RR and the signs of respiratory distress is mandatory. Interestingly, nurses were found to not effectively measure RR as all patients documented had identical RR on the vitals charts.

### Insights gained on treatment

The use of accuhalers with complicated devices posed a problem for our patients with neurocognitive impairment; often, a carer was required to help administer the dose. Nebulisers were not permitted in our wing because of the danger of aerosolising COVID particles. After prolonged use of nasal cannula with high oxygen flow rates, adequate lubrication of the nose with sesame oil drops or saline spray helped ease the crusty and dry nasal mucosa.

Anti-pyretics and analgesics such as paracetamol resulted in constipation, and the awareness that regular bowel habits had to be maintained, as constipation, faecal impaction and urinary tract infection contributed to delirium, should be created.

Transient dysphagia with COVID and dysphagia generally in advanced dementia were nursing challenges. Patients and healthcare workers were encouraged to maintain oral feeds. Naso-gastric tube feeding was not an option. Patients required supplemental protein-based shakes. Oral care was, unfortunately, often forgotten. Staff had to be reminded to use a saline rinse or gauze dipped in warm saline to maintain good oral care.^16^ The use of a toothbrush was not permitted to avoid aerosolising of COVID particles. Some residents developed oral thrush from the inhalers and nystatin drops were administered.

The British Geriatric Society described a process called ‘physical deconditioning’: reduced mobility, falls, increased confusion, loss of appetite, new swallowing problems, weight loss, constipation, worsening incontinence, drowsiness and becoming withdrawn after COVID infection.^17^ Studies are now identifying delirium post COVID.^2^ There was an active drive to maintain as much of the ‘normal frail care environment’ into the isolation unit as possible. This included certain foods, smells, music and certain voices that the residents were accustomed.

## In conclusion

In conclusion, the following lessons were learned regarding the management of an outbreak in a care home: (1) do not underestimate the emotional toll that the virus has on both the patient and the staff working in the environment and accept that some healthcare workers may not be comfortable working under such circumstances; (2) patients with severe neurocognitive impairment lack the capacity to understand the importance of using NPO and compliance is an issue; (3) monitoring of vitals is crucial for optimal management of patients, and continuous education of staff (day and night shifts) about the importance of doing vitals 6–8 hourly and associated markers of delirium is important; (4) knowledge of patients baseline frailty by using the Clinical Frailty Scale should be assessed *before* the pandemic. This frailty assessment will help the recovery process and guide the multi-disciplinary team towards returning the patient to their baseline; (5) hydration is crucial, especially in neurocognitive impaired patients who are unable to drink without assistance; (6) the availability of oxygen and an onsite medical doctor allowed *in situ* care of over 50% of these complex patients. This potentially alleviates the burden on hospital facilities which generally are not able to focus on the specific care needs of these vulnerable patients; (7) reduction of patient anxiety by creating a safe, familiar environment such as by playing background music and having familiar staff around; (8) giving families the opportunity to see their loved ones, even through a glass window, with hand written love messages; (9) encourage residents in nursing homes to have an advanced directive and advanced care planning; (10) create family WhatsApp groups with regular updates regarding their loved ones.

Thus, in conclusion, the aim of the practical recommendations mentioned in this protocol will assist practitioners to better manage frail older persons which are COVID-19-positive regardless of the clinical setting. Because of the small number of cases and inadequate validation, this protocol represents a practical recommendation for the care of older persons which are COVID-19-positive reaffirming that these most vulnerable older persons receive adequate and appropriate care within their familiar environments as safely possible.

## Appendix 1

**TABLE 1-A1:** Recommended treatment protocol for coronavirus disease 2019 positive geriatric patient within an old age home.

**Preventative measures:** Restriction of visitors and non-essential staff. Screening of staff for COVID-19 symptoms via twice daily measurement of body temperature. Personal protective equipment to be worn by staff including mask, apron, visor and gloves. Social distancing.
**Measures for monitoring all residents:** Twelve-hourly measurement: Blood pressure, pulse rate, temperature, oxygen saturation and delirium. Identify comorbidities: Frailty, hypertension, cerebrovascular disease, diabetes, cardiovascular disease or obesity. Prepare advanced directives.
**The first positive case:** Isolate the patient and all nursing contacts immediately. Separate the isolation unit from the main facility with separate amenities and resources. Avoid staff’s unnecessary contact with positive cases.
**Monitoring positive patients:** Four- to six-hourly measurement: Blood pressure, pulse rate, temperature, oxygen saturation and delirium Hydration Vitamins: Vitamin D (Calciferol) one daily po for 3 days Zinc tablet one daily po or zinc lozenges Thiamine 100 mg daily po Vitamin C 500 mg twice daily po.
**Treatment:** NPO 2–6 L oxygen (supplemental). Inhalers: Salmeterol/fluticasone 1–2 inhalations 6 hourly. Cortisone: Methylprednisone 40 mg IMI for 3 days. Antoicoagulant: Enoxaparin 20 mg s/c daily or rivaroxaban 10 mg daily. Antibiotics: Azithromycin 500 mg daily for 3 days po or ceftriaxone 1 g IMI 24 hourly (dilute with 1% lignocaine) for 5 days. Anti-pyretic: Paracetamol 1–2 tablets or 15–20 mL syrup 6 hourly. Deprescribe: Angiotensin converting enzyme inhibitors, diuretics and anti-inflammatories. Physio: Maintain in the prone position; mobilise to chair and gentle bed mobility/to toilet as tolerated by patient. Do not nebulise or do active chest percussions.
**Reasons for admission:** Haematological: D-dimer > 10; CRP > 150; acute kidney injury. Not maintaining oxygen > 90% on 5 L NPO2. Chronic delirium. Family request or no advanced directive.

NPO, nasal prong oxygen; CRP, C-reactive protein; IMI, intramuscualar injection.
